# Retrospective analysis of the clinical features of 12 cases of MOG antibody-associated aseptic meningitis

**DOI:** 10.3389/fmed.2026.1729513

**Published:** 2026-02-16

**Authors:** Xiufang Ding, Yanli Cao, Ruifeng Jin, Yong Liu, Juan Li, Chunmei Yu

**Affiliations:** 1 Department of Neurology, Children’s Hospital Affiliated to Shandong University, Jinan, Shandong, China; 2 Department of Neurology, Jinan Children’s Hospital, Jinan, Shandong, China

**Keywords:** anti-myelin oligodendrocyte glycoprotein antibody-associated diseases, demyelination, meningitis, MOG, unexplained fever

## Abstract

**Background:**

In recent years, MOG antibody-associated aseptic meningitis has gradually been reported, but its clinical features are not yet well defined.

**Methods:**

A retrospective analysis was conducted on 67 children diagnosed with MOG antibody-associated diseases and treated at the Affiliated Children’s Hospital of Shandong University from January 2021 to June 2024. Out of these cases, 12 exhibited aseptic meningitis. The key features of their clinical manifestations, blood cell analysis, erythrocyte sedimentation rate (ESR), C-reactive protein (CRP) levels, cerebrospinal fluid (CSF)-related tests, and cranial MRI results were summarized for these 12 cases.

**Results:**

A total of 12 cases (100%) presented with prolonged fever as the main clinical feature. Routine blood tests revealed the following: white blood cell counts ranging from 15.92 to 34.0 × 10^9^/L; neutrophil ratios of 76.5 to 90.3%; ESR ranging from 48 to 105 mm/h; and CRP ranging from 21.6 to 116.0 mg/L. The CSF white blood cell counts ranged from 15 to 182 × 10^6^/L. Cranial MRI revealed inflammatory changes in four cases. The MOG antibody titer was 1:100 in 5 cases, 1:32 in 5 cases, and 1:320 in 2 cases. After glucocorticoid therapy, the body temperatures of these children normalized, and the follow-up routine blood test, CRP, and ESR results gradually returned to the respective normal ranges.

**Conclusion:**

These findings highlight that MOG antibody-associated aseptic meningitis primarily manifests as prolonged fever, with peripheral inflammatory markers that mimic a severe bacterial infection, while CSF profiles resemble those of viral encephalitis. Clinicians should consider MOG antibody-associated disorders in children with unexplained recurrent fever.

## 1 Introduction

Fever of unknown origin (FUO) in children lacks a consistent definition. A recent study defined pediatric FUO as a persistent fever lasting ≥8 days, with at least one daily temperature measurement >38.0 °C, and no diagnosis established after initial outpatient or inpatient evaluation, including detailed history, comprehensive physical examination, and preliminary laboratory assessments (1). This condition is commonly associated with infectious diseases, connective tissue disorders, and neoplasms. However, some children remain difficult to classify into these traditional categories even after systematic investigations.

MOG antibody-associated disease (MOGAD) is a group of central nervous system inflammatory demyelinating disorders mediated by anti-MOG IgG antibodies. Its typical clinical phenotypes include optic neuritis, acute myelitis, and acute disseminated encephalomyelitis. In recent years, the disease spectrum of MOGAD has expanded to include conditions such as cortical encephalitis (2), cranial nerve involvement (3), and even aseptic meningitis presenting with meningitis-like symptoms (4). Among these, MOG antibody-associated aseptic meningitis (MOG-AAM), characterized predominantly by prolonged fever, warrants particular attention. Affected children often exhibit markedly elevated systemic inflammatory markers, which can lead to misdiagnosis as severe bacterial infection, resulting in unnecessary broad-spectrum antibiotic use and delayed diagnosis. However, there is still a lack of large-scale studies both domestically and internationally on the systematic clinical characteristics of MOG-AAM presenting with isolated prolonged fever as the initial or predominant manifestation. This gap contributes to difficulties in clinical recognition and delays in treatment. Through a retrospective analysis of clinical data from 12 diagnosed pediatric cases, this study aims to systematically summarize their clinical manifestations, laboratory and imaging findings, and preliminarily explore their treatment responses and prognosis. The goal is to establish key clues for the early identification of this disease and to provide new diagnostic perspectives for the etiological differentiation of fever of unknown origin in children.

## 2 Subjects and methods

### 2.1 General information

Clinical data of pediatric patients diagnosed with MOGAD who were hospitalized at the Children’s Hospital Affiliated to Shandong University from January 2021 to June 2024 were collected. The inclusion criteria were as follows: MOG-AAM, diagnosed in accordance with the international diagnostic criteria for MOGAD (5) and the diagnostic criteria for aseptic meningitis (6). To more accurately summarize the clinical characteristics of MOG-AAM and avoid the influence of other confounding factors, this study excluded cases with the following concurrent diseases prior to inclusion: (1) presence of comorbidities, such as otitis media, tonsillitis, pneumonia, appendicitis, leukemia, or tumors; (2) previous diagnosis of uncontrolled epilepsy, leukoencephalopathy, brain injury, or any other neurological disease; (3) previous diagnosis of autoimmune diseases, such as Hashimoto’s thyroiditis or systemic lupus erythematosus; (4) consumption of immunosuppressants or presence of an immune deficiency disease in the 3 months prior to admission; (5) MOG antibody titer <1:10; (6) failure to meet the diagnostic criteria for meningitis. Accordingly, 12 children were finally included in the present study. All of these 12 children were previously healthy with normal growth and developmental milestones and no family history of the disease. The study was approved by the Ethics Committee of the Children’s Hospital Affiliated to Shandong University (Approval No. SDFE-IRB/T-2025038).

### 2.2 Methods

A retrospective analysis was conducted of the clinical data of the 12 hospitalized children who initially presented with prolonged fever and were finally diagnosed with MOG-AAM. All 12 children underwent comprehensive examinations, including routine blood tests, ESR, CRP, Epstein–Barr virus (EBV), Brucella, fungi, blood cultures, tuberculin tests, levels of antinuclear antibodies, complements, anti-neutrophil cytoplasmic antibodies, immunoglobulins, and T lymphocyte subsets, bone marrow punctures, lumbar punctures, high-throughput gene sequencing for blood and/or CSF pathogens, levels of autoimmune encephalitis antibodies (CBA, Hangzhou CredMed Diagnostics Co., Ltd), chest and abdominal ultrasound or CT, cardiac ultrasound, and cranial MRI. Bone marrow cytology analysis was independently performed by two experienced clinical laboratory physicians. The analysis provided both quantitative data, including the percentages of major cell lineages and the myeloid-to-erythroid (M:E) ratio, and qualitative morphological assessment, noting features such as cell maturity and dysplasia. All slides were examined in a blinded fashion, and any discrepancies between the two assessors were resolved through a joint re-evaluation to reach a consensus.

### 2.3 Statistical analysis

Statistical analysis was performed using SPSS 26.0. Discrete data were expressed as numbers and percentages. Owing to the small sample size and wide ranges of continuous variables (e.g., white blood cell count and CRP), which were expected to be non-normally distributed, all continuous data were analyzed using descriptive statistics (range, median).

## 3 Results

### 3.1 Symptoms and signs

The included children comprised 5 boys (41.7%) and 7 girls (58.3%), with a median age of 6.915 years. All children experienced fever (100%), with 9 cases (75%) having mild headaches during the fever, which relieved after the fever subsided. In addition, 2 others among the 12 cases (16.7%) experienced vomiting, 1 case (8.3%) experienced mild pain in the neck, limbs, and abdomen during fever, and 1 case (8.3%) had chills. Focal neurological deficits or the involvement of any other organ were not observed. Physical examinations revealed good mental status in all 12 children (100%), with mild neck stiffness in 5 cases (41.7%) and a positive Babinski sign in 1 case (8.3%). No other positive signs were noted.

### 3.2 Auxiliary examinations

The white blood cell counts (15.92–34.00 × 10^9^/L; median 23.615 × 10^9^/L), neutrophil ratios (76.5–90.3%; median 79.25%), ESR (48–105 mm/h; median 65 mm/h), CRP levels (21.6–116.0 mg/L; median 48.25 mg/L), and cerebrospinal fluid white blood cell counts (15–182 × 10^6^/L; median 71 × 10^6^/L) were recorded in the 12 pediatric patients. Their serum MOG antibody titers ranged from 1:32 to 1:320. Some of them also had cranial MRI lesions. The children were negative for autoimmune encephalitis antibodies (excluding MOG). One case was positive for EBV DNA, and the levels of *Mycoplasma pneumoniae* antibody IgM were elevated in 1 case. Negative results were also obtained in Brucella and fungi measurements, blood cultures, tuberculin tests, and pathogen gene sequencing for blood and/or CSF. The analysis of immunoglobulins and T lymphocyte subsets revealed no significant abnormalities. The analysis of antinuclear antibodies, complement levels, and anti-neutrophil cytoplasmic antibodies, among other immune markers, presented negative results. Bone marrow cytology indicated infectious marrow. Chest and abdominal ultrasound (or CT) revealed no significant abnormalities. Cardiac ultrasound revealed no coronary dilation or any other lesions.

### 3.3 Treatment outcomes and follow-up

Case 1 (Index Patient): The index case, admitted to the Children’s Hospital Affiliated to Shandong University, was initially treated with ceftriaxone, acyclovir, dexamethasone, and mannitol. Recurrent fever developed 4 days after dexamethasone discontinuation. Antibiotic therapy was then escalated to meropenem plus vancomycin, yet intermittent fever persisted. On day 35 of the disease course, a follow-up cranial MRI revealed inflammatory lesions in the thalamus and cerebellum. Subsequent workup was positive for MOG antibodies, confirming a diagnosis of MOGAD. The patient was subsequently treated with intravenous immunoglobulin (IVIG) and methylprednisolone pulse therapy. At discharge, laboratory tests revealed normalization of the complete blood count, ESR, and CRP, and cranial MRI showed partial resolution of the intracranial lesions. The patient was started on a 3-month tapering course of oral prednisone. After a 4-month remission, the disease relapsed with symptoms of fever, slurred speech, and seizures. At relapse, the MOG antibody titer was elevated. The patient was re-treated with IVIG and methylprednisolone pulse therapy, followed by a 6-month tapering course of oral prednisone, in combination with mycophenolate mofetil. No further relapse has occurred during the 2 years of follow-up.

Cases 2–12: The remaining 11 children all received empirical antibiotic therapy upon admission (e.g., ceftriaxone, mezlocillin, or meropenem combined with vancomycin). Serum MOG antibody testing was performed concurrently. Once MOG antibody positivity was confirmed, treatment was switched to IVIG combined with either methylprednisolone or dexamethasone. After approximately 2 weeks of hospitalization, follow-up assessments showed marked improvement or normalization in complete blood count, ESR, CRP, cerebrospinal fluid analysis, and cranial MRI findings. Ten patients received a tapering course of oral prednisone for 3 months, and one patient received it for 4 months. Among these patients, one relapsed 6 months after completing the 3-month prednisone course, presenting with an elevated MOG antibody titer and new subcortical white matter abnormalities on MRI. Following re-initiation of immunosuppressive therapy, this patient has remained relapse-free during 2 years of follow-up. The other 10 patients have been followed for more than 1.5 years (exceeding 2 years in 8 cases), with no recurrences.

## 4 Discussion

Unexplained fever in children is commonly associated with infections, connective tissue diseases, malignancies, and other etiologies (1). At the Children’s Hospital Affiliated to Shandong University, 12 pediatric patients meeting the diagnostic criteria for fever of unknown origin (7) underwent a comprehensive diagnostic workup. All patients exhibited significantly elevated white blood cell counts, ESR, and CRP levels, suggestive of severe bacterial infection. However, no improvement in these inflammatory markers was observed despite empiric broad-spectrum antibiotic therapy with meropenem and vancomycin. Pathogenic investigations identified EBV and *Mycoplasma pneumoniae* infections in two cases; however, these patients did not display typical clinical manifestations of these infections (e.g., rash, hepatic dysfunction, cough, or lobar pneumonia). Other pathogen tests for *Brucella*, fungi, and blood cultures were negative. Tuberculin skin tests were negative, and high-throughput microbial gene sequencing of blood and/or CSF failed to reveal significant pathogens, thereby excluding severe bacterial or atypical infections. Immunological evaluations showed negative results for antinuclear antibodies, complement levels, rheumatoid factor, and anti-neutrophil cytoplasmic antibodies. Cardiac ultrasonography revealed no coronary artery dilatation, and all patients lacked clinical features such as rash, arthralgia, or lymphadenopathy. Consultation with pediatric rheumatology specialists ruled out connective tissue diseases, including juvenile rheumatoid arthritis, Kawasaki disease, and subacute necrotizing lymphadenitis. Bone marrow cytology demonstrated an infectious marrow pattern, while abdominal/thoracic ultrasonography (or CT) revealed no abnormalities. No constitutional symptoms (e.g., weight loss or fatigue) suggestive of hematologic malignancies or solid tumors were observed. Immunoglobulin levels and T-lymphocyte subsets remained within reference ranges, excluding immunodeficiency disorders. Notably, all patients exhibited elevated CSF leukocyte counts, and some had cranial MRI lesions. Serum anti-MOG antibody titers were elevated, supporting the diagnosis of MOGAD.

MOG, a glycoprotein exclusively expressed in the central nervous system (CNS), is the target of pathogenic autoantibodies (8) that mediate inflammatory demyelinating disorders of the CNS. These conditions are collectively termed MOGAD, with classic phenotypes including optic neuritis, acute transverse myelitis, and acute disseminated encephalomyelitis. Recently, an increasing number of atypical clinical phenotypes have been reported, such as cortical encephalitis (2), overlapping syndromes (9), oculomotor neuritis (3), and MOG antibody-associated myelitis with normal spinal MR (10), ocular flutter/opsoclonus myoclonus, chronic lymphocytic inflammation with pontine perivascular enhancement responsive to steroids, and so on (11). MOG antibody-associated meningitis was first reported in 2019 (12). According to the international diagnostic consensus on MOG antibody-associated diseases (5) and the diagnostic criteria for aseptic meningitis (6), all 12 children analyzed in the present study met the diagnostic criteria for MOG-AAM.

Studies show that prolonged fever is a clinical phenotype of MOGAD and could be used as a differential diagnosis for unexplained fever (13). All 12 children analyzed in the present study had prolonged fever as the main clinical manifestation, with good mental status throughout the illness. Antibiotic treatment was ineffective in all children, although the children were sensitive to glucocorticoid therapy. All children had elevated white blood cell counts, ESR, and CRP. Most children had no significant abnormalities on brain MRI, which is consistent with the characteristics reported in previous studies (13); however, in this study, all 12 pediatric cases showed alterations in CSF cell counts. In terms of treatment, compared to the classical MOGAD pulse therapy with methylprednisolone, the children with MOGAD presenting mainly with prolonged fever achieved good treatment outcomes with low-dose intravenous dexamethasone followed by oral prednisone. It should be noted that, as a retrospective study with a limited sample size, the efficacy of the low-dose steroid regimen still requires further investigation. Nevertheless, early steroid intervention may better support recovery and mitigate recurrence. The exact mechanisms underlying fever and elevated inflammatory markers in MOGAD remain unclear. Studies demonstrated elevated cerebrospinal fluid cytokines, particularly interleukin-6 (IL-6) and granulocyte colony-stimulating factor (G-CSF), in MOGAD patients (14, 15). IL-6, a pro-inflammatory cytokine (16), induces prostaglandin release, which acts on the hypothalamus to elevate the body’s temperature set point, thereby triggering fever. Both G-CSF and IL-6 stimulate bone marrow progenitor cells, leading to increased white blood cell counts and elevated inflammatory markers (15).

Regrettably, we did not include a control group of children with MOG antibody-negative aseptic meningitis (e.g., viral meningitis, subacute necrotizing lymphadenitis with cerebrospinal fluid abnormalities, or Kawasaki disease with cerebrospinal fluid abnormalities) for comparison. However, viral meningitis typically follows a benign, self-limiting course and rarely causes recurrent fever. Connective tissue diseases (such as subacute necrotizing lymphadenitis and Kawasaki disease) may induce prolonged fever with mild cerebrospinal fluid abnormalities. Notably, most children with subacute necrotizing lymphadenitis do not exhibit hematological markers (blood tests, ESR, and CRP) characteristic of severe bacterial infections, which contrasts with the clinical features observed in MOG-AAM. Conversely, Kawasaki disease patients with cerebrospinal fluid abnormalities may display hematological parameters resembling severe bacterial infections (elevated ESR and CRP) and viral encephalitis-like cerebrospinal fluid changes. However, such cases predominantly affect children under 5 years old with persistent fever, typically accompanied by classic Kawasaki disease manifestations like bulbar conjunctival congestion, indurative edema or desquamation of the hands and feet, rashes, and lymphadenopathy. To facilitate clinical differentiation, Table 1 summarizes the key distinguishing features among MOG-AAM, viral meningitis, subacute necrotizing lymphadenitis (Kikuchi disease), and Kawasaki disease.

**Table 1:** Key features distinguishing MOG-AAM from other causes of aseptic meningitis.

Characteristic	MOG-AAM	Viral meningitis	Kikuchi disease	Kawasaki disease
Inflammatory markers	Markedly elevated (WBC, ESR, CRP)	Mildly elevated or normal	Normal or mildly elevated	Elevated
CSF findings	Mild pleocytosis (viral-like)	Lymphocytic pleocytosis	Not typically performed	Normal
Response to antibiotics	No response	No response	No response	No response
Response to steroids	Dramatic response	Not required	Variable	Not typically required
Clinical course	Requires immunotherapy	Self-limiting	Self-limiting	Self-limiting, but requires treatment
Risk of recurrence	16.7% (in our cohort)	Rare	Rare	Rare
Long-term complications	Risk of demyelinating events	None	None	Coronary artery complications
Characteristic feature	Preserved mental status, no typical neurological signs	Viral prodrome, self-limiting	Lymphadenopathy, no severe inflammatory markers	Mucocutaneous features, age <5 years
Diagnostic test	Serum MOG antibody	Viral PCR/serology	Lymph node biopsy	Clinical criteria + echocardiography

In summary, MOG-AAM presenting with prolonged fever can be differentiated from other conditions based on several key characteristics. Unlike self-limiting viral meningitis, MOG-AAM demonstrates marked peripheral inflammatory marker elevation (resembling severe bacterial infection) with dramatic steroid-sensitivity but antibiotic-resistance. While Kikuchi disease may cause prolonged fever, it typically presents with prominent lymphadenopathy without the severe inflammatory marker elevation seen in MOG-AAM (17). Although Kawasaki disease shares the pattern of markedly elevated inflammatory markers, it predominantly affects younger children with characteristic mucocutaneous manifestations absent in MOG-AAM (18). It is particularly important to note that MOG-AAM, like anti-MOG antibody-associated disorders, carries a risk of relapse. Long-term follow-up is necessary. In contrast, viral meningitis and Kikuchi disease are typically self-limited conditions (17). Thus, the key distinguishing features of MOG-AAM include significantly elevated inflammatory markers, responsiveness to corticosteroid therapy, and risk of recurrence.

Notably, these pediatric MOG-AAM patients often presented with preserved mental status and a striking lack of classic neurological symptoms, which is consistent with previous research (19). Since the diagnosis of MOGAD traditionally depends on identifying “core demyelinating events” like optic neuritis or transverse myelitis (5), a key clinical question arises: how do doctors commonly predict MOGAD in a patient presenting with fever and systemic inflammation, yet without apparent neurological signs? To address this challenge, we propose a novel diagnostic concept termed “clinical-laboratory dissociation” for the early identification of MOG-AAM, defined by the following three distinct features: (1) clinical dissociation: marked fever and elevated inflammatory markers coexist with preserved mental status, in the absence of characteristic signs of other systemic diseases; (2) inflammatory pattern dissociation: peripheral blood shows a severe “bacterial infection-like” inflammatory profile, whereas cerebrospinal fluid exhibits only mild “viral encephalitis-like” changes; (3) treatment response dissociation: broad-spectrum antibiotics are ineffective, whereas glucocorticoids demonstrate remarkable sensitivity. Therefore, when clinicians encounter a child with recurrent fever, preserved mental status, markedly elevated systemic inflammatory markers, viral encephalitis-like CSF changes, and poor response to antibiotics, the possibility of MOG-AAM should be considered regardless of cranial MRI findings, and serum MOG antibody testing is recommended. This approach not only helps avoid unnecessary antibiotic use and prevent diagnostic delays but, more critically, enables the initiation of immunomodulatory therapy before the onset of typical neurological damage, thereby potentially modifying the disease course and reducing relapse risk.

The existing literature, both domestic and international, on MOGAD with prolonged fever is scarce. Several front-line clinicians, therefore, lack awareness of this condition, which often leads to delayed diagnosis. In the present study, a literature search was conducted using MOG, fever, and aseptic meningitis as keywords on PubMed, Web of Science, Google Scholar, CNKI, and the Chinese Medical Journal Full-text Database. The search revealed that MOG-AAM is increasingly being reported (4, 19–23), with over 90% of the patients reported to have a fever. Building on a summary of the main features of such patients—including prolonged fever, significantly elevated inflammatory markers, sensitivity to corticosteroid therapy, and risk of recurrence—this study further proposes the key diagnostic clue of “clinical-laboratory dissociation” (encompassing dissociation in clinical presentation, inflammatory patterns, and treatment response), thereby providing a more actionable diagnostic approach for early identification of the disease, facilitating early intervention before the onset of typical neurological injury and reducing inappropriate treatment and clinical delays.

Previous literature has reported rare cases of MOGAD primarily presenting with prolonged fever. Due to limited clinical experience, the index case was misdiagnosed and received prolonged antibiotic therapy. The detection of MOG antibodies was only pursued after the appearance of inflammatory lesions on brain MRI, leading to delayed diagnosis and consequent postponement of appropriate treatment. Based on this experience, MOG antibody testing was actively performed for the subsequent 11 children in the cohort upon admission. Following a positive result, most patients were treated with a relatively low-dose dexamethasone regimen, followed by a sequential three-month oral prednisone tapering schedule. This treatment strategy differs from the high-dose methylprednisolone pulse therapy commonly employed for other MOGAD phenotypes (24). All patients demonstrated a favorable response to immunomodulatory therapy. The apparent efficacy of the low-dose regimen in this cohort may reflect the disease characteristics of MOG-AAM, characterized by prolonged fever, which appears to manifest predominantly with systemic rather than neurological involvement.

Recurrence was observed in two patients (16.7%), underscoring the importance of identifying risk factors to guide long-term management. Although limited by the sample size, several potential clues were noted. First, in the index case, the interval from symptom onset to diagnosis was 35 days, and subsequent relapse occurred. Combined with literature (25), we speculate that the delay in initiating specific immunotherapy may be associated with an increased risk of long-term relapse. Second, consistent with previous studies (26), disease relapses were consistently accompanied by a significant increase in serum MOG antibody titers. This suggests that longitudinal monitoring of antibody titers may serve as a biomarker for assessing disease activity and predicting the risk of recurrence. Furthermore, the emergence of new abnormal MRI signals (e.g., subcortical white matter lesions) during relapse aligns with the literature (27), underscoring the value of imaging for monitoring disease activity. Based on these observations, we speculate that elevated MOG antibody titers, treatment delays, and the emergence of new MRI lesions may be important factors indicating an increased risk of recurrence.

All patients in this study were followed up for a minimum of 1.5 years, with the majority (10/12, 83.3%) completing over 2 years of observation. During this period, two patients experienced relapse, both occurring within the first year after discontinuation of initial treatment. This suggests that the risk of relapse in MOG-AAM is elevated early after treatment discontinuation, particularly within the first year, aligning with the recognized relapse pattern of MOGAD (28). Therefore, we recommend systematic follow-up for children with MOG-AAM for at least 2 years. Special attention should be paid to the first year after stopping treatment, as this period poses a high risk for relapse. For patients with the aforementioned high-risk factors for recurrence, closer integrated monitoring with clinical, imaging, and serological tests should be provided.

Given the small number of cases and the retrospective nature of this study, key issues such as the minimum effective dose of corticosteroids, relapse risk (including its predictors), and long-term prognosis remain unclear. Future work should expand MOG antibody screening in children with FUO or aseptic meningitis and conduct prospective, large-scale studies to systematically characterize the clinical features of this condition and resolve these issues.

In summary, our study provides important clinical insights. It establishes a practical “clinical-laboratory dissociation” framework for early recognition of MOG-AAM presenting with prolonged fever, which is often misdiagnosed as bacterial infection. It expands the clinical spectrum of MOGAD to include predominantly systemic manifestations, thereby adding a new diagnostic perspective to pediatric fever of unknown origin. Early recognition enables timely immunotherapy rather than prolonged antibiotic use, while identified relapse risk factors guide personalized follow-up strategies. These findings can improve diagnostic accuracy, reduce treatment delays, and enhance outcomes for children with this challenging MOGAD presentation.

## 5 Conclusion

Our study may identify key advances in MOGAD: (1) defining MOG-AAM as a distinct clinical subtype marked by prolonged fever and inflammatory dysregulation, expanding MOGAD’s phenotypic spectrum; (2) establishing MOG-AAM as a novel diagnostic entity to reduce delays and misdiagnosis in pediatric unexplained fever; and (3) MOG-AAM may be an initial clinical manifestation of typical MOGAD. If untreated, it could potentially progress to core demyelinating events; however, early immunomodulatory therapy might help slow disease progression and lead to better clinical outcomes.

## Data Availability

The datasets presented in this article are not readily available because of ethical and privacy restrictions. Requests to access the datasets should be directed to the corresponding author.
